# Quantum Algorithm for Variant Maximum Satisfiability †

**DOI:** 10.3390/e24111615

**Published:** 2022-11-05

**Authors:** Abdirahman Alasow, Peter Jin, Marek Perkowski

**Affiliations:** Department of Electrical & Computer Engineering, Portland State University, Portland, OR 97207, USA

**Keywords:** satisfiability, maximum satisfiability, quantum counter, Grover search algorithm, quantum circuit

## Abstract

In this paper, we proposed a novel quantum algorithm for the maximum satisfiability problem. Satisfiability (SAT) is to find the set of assignment values of input variables for the given Boolean function that evaluates this function as TRUE or prove that such satisfying values do not exist. For a POS SAT problem, we proposed a novel quantum algorithm for the maximum satisfiability (MAX-SAT), which returns the maximum number of OR terms that are satisfied for the SAT-unsatisfiable function, providing us with information on how far the given Boolean function is from the SAT satisfaction. We used Grover’s algorithm with a new block called quantum counter in the oracle circuit. The proposed circuit can be adapted for various forms of satisfiability expressions and several satisfiability-like problems. Using the quantum counter and mirrors for SAT terms reduces the need for ancilla qubits and realizes a large Toffoli gate that is then not needed. Our circuit reduces the number of ancilla qubits for the terms T of the Boolean function from T of ancilla qubits to $\approx \log_{2}T+1$. We analyzed and compared the quantum cost of the traditional oracle design with our design which gives a low quantum cost.

## 1. Introduction

### 1.1. Satisfiability

The satisfiability (SAT) problem for a given Boolean function is the problem of determining if there exists a set of assignment values of input variables for the given Boolean function that evaluates this function to TRUE. Boolean or propositional-logic expressions are formed using operators AND, OR, EXOR, and NOT from input variables. Satisfiability expression (circuit) is often expressed as a product-of-sum (POS) form. POS is a logical ANDs of OR terms, where each OR term is an inclusive sum of literals. For instance, the POS SAT function $f\left(a,b,c\right)=\left(a+b+\bar{c}\right)\left(\bar{a}+\bar{b}+c\right)\left(b+c\right)$ is satisfiable because when c=1 and either a or b is equal to 1, then f(a,b,c) evaluates to 1. Another example, $f\left(a,b\right)=\left(a+b\right)\left(\bar{a}+\bar{b}\right)\left(\bar{a}+b\right)\left(a+\bar{b}\right)\;$ is not satisfiable because no binary assignment of values for variables a and b, f(a,b) would evaluate to 1.

Satisfiability problems have a wide range of applications, such as model checking in electronic design automation (EDA) [1], automatic test pattern generation (ATPG) [2], software and hardware verification [3], and circuit design [4]. Satisfiability problems also have many applications in Artificial Intelligence [5], robotics, and electronic design. Based on Cook’s theorem [6], satisfiability is an NP-complete problem. Solving a satisfiability problem involving many variables and terms using traditional algorithms is computationally expensive.

### 1.2. Maximum Satisfiability

Maximum satisfiability (MAX-SAT) is an optimization version of the SAT problem. MAX-SAT finds the maximum number of constraints of a given Boolean function that are satisfied. Suppose a Boolean function in the POS form contains thousands of sum (OR) terms (also called clauses). The MAX-SAT problem is to examine the maximum number of terms that are satisfied. For example, $f\left(a,b,c,\;\ldots ,\;N\right)=\left(a+b+\bar{c}\right)\left(\bar{a}+\bar{b}+c\right)\left(b+c\right)\ldots \left(\ldots ,\;\ldots \right)=1$. The function f is true for a binary assignment of values to variables a,b,c, …, N for which all terms are true. This is the SAT satisfiability. In contrast, the goal of MAX-SAT is not only to find the decision satisfied/unsatisfied (yes/no) but also to provide the maximum number of terms (clauses) that are satisfied with the actual satisfying assignment values for the variables in case the formula is not SAT satisfiable. The MAX-SAT is considered to be an NP-hard problem [7]. 

There are several extensions and modifications to the MAX-SAT problem formulated as above. For instance, sometimes not all constraints of a problem can be satisfied, but some of them must be satisfied. In such a case, MAX-SAT constraints can be divided into two sets of clauses:

- Hard clauses: The constraints that must be satisfied.
- Soft clauses: The constraints that may or may not be satisfied, but we want to satisfy as many as possible.

There are three main variants of MAX-SATs [8,9]:

1. Weighted MAX-SAT: Each clause has an associated weight cost, and the objective is to maximize the sum of the weights of the satisfied clauses.
2. Partial MAX-SAT: Finds the assignment values for the variables that must be satisfied for all hard clauses and must be maximized on the soft clauses.
3. Weighted partial MAX-SAT is a combination of the partial and weighted MAX-SAT.

The applications of these different variants will be discussed in the next section.

## 2. Related Work

### 2.1. Maximum Satisfiability Applications

There are many optimization problems and real-world applications that can be encoded to MAX-SAT. Some of the successful applications used for MAX-SAT are data analysis and machine learning, planning and scheduling, verification and security, bioinformatics, and combinatorial optimization [8]. We will briefly discuss some of these applications.

#### 2.1.1. Data Analysis and Machine Learning

MAX-SAT has been used in many problems in Data Analysis, Artificial Intelligence (AI) and Machine Learning [10]. Correlation clustering is a well-studied problem in data analysis and AI in which data are divided into subgroups in a meaningful way. Discovering an optimal way of making such a division is a computational challenge. There are many approaches to find the optimal clustering, including a greedy local-search and approximation algorithms, which cannot find optimal clusterings. Solving exact formulations of the correlation clustering as MAX-SAT based approach leads to cost-optimal correlation clustering [11]. Bayesian Network Structure Learning (BNSL) is a computationally hard problem of finding a directed acyclic graph structure that optimally describes a given data structure. These problems use learning that can be based on probabilistic or exact inference methods. Using MAX-SAT as exact inference has been shown to yield a competitive approach to learning optimal bounded tree-width Bayesian network structures (BTW-BNSL) [12]. There are many other AI applications and data analysis approaches formulated as MAX-SAT, including causal structure discovery [13], and deriving interpretable classification rules [14].

#### 2.1.2. Planning and Scheduling

MAX-SAT can be applied in linear temporal logic (LTL) specifications for robotic motion planning and control of autonomous systems. Suppose that we want to design a controller for a robotic museum guide; the robot has to give a tour of the exhibitions in a specific order, which constitutes the hard specification. Preferably, it also avoids certain locations, such as the staff’s office, the library, or the passage when it is occupied. These preferences are encoded in the soft specifications [15]. This is an example of a partial MAX-SAT formulation. There are other planning problems that can be encoded as MAX-SAT for cost-optimal planning [16,17]. 

Scheduling problems are well-known problems that appear in various contexts, including health care, airlines, transportation services, and various financial and money transfer problems in organizations. These scheduling problems can be encoded as a weighted partial MAX-SAT problem [18]. 

#### 2.1.3. Verification and Security

Functional verification tasks dominate the effort of contemporary VLSI and SoC design cycles. A major step of functional verification is design debugging, which determines the root cause of failed verification tasks such as simulation or equivalence checking. The MAX-SAT formulation is used as a pre-processing step to construct a highly optimized debugging framework [19,20,21]. One of the techniques for debugging both hardware and software is fault localization, where the goal is to pinpoint the localization of bugs. Fault localization is performed using the MAX-SAT approach to reduce and improve automation for error localization, which can speed up the debugging process [22,23].

MAX-SAT has many applications in security. Starting with solving the user authorization query problem [24], reconstructing AES key schedule images [25], detecting hardware Trojans [26], and malware detection [27].

#### 2.1.4. Bioinformatics

MAX-SAT has many applications in the bioinformatics field, such as cancer therapy, finding the optimal set of drugs to fix or rectify the fault areas of the gene regulatory network [28], modeling biological networks and checking their consistency [29], finding the maximum similarity between RNA sequences [30] and finding the minimum-cardinality set of haplotypes that explains a given set of genotypes [31].

#### 2.1.5. Combinatorial Optimization Problems

Combinatorial optimization problems are widely studied in fundamental academic research and in solving real-life problems. Many of these problems are NP-hard, where an exhaustive search is not tractable. For instance, MAX-SAT has been used to encode and solve such problems as the Max-Clique problem [32,33,34], given a group of vertices. The maximal clique is the largest subset of vertices in which each point is directly connected to every other vertex in the subset. 

Other applications within this domain that have been encoded into MAX-SAT consist of determining the Treewidth of a graph [35] and finding solutions for the maximum quartet consistency problem [36].

### 2.2. Classical Algorithm for Maximum Satisfiability Problem 

There are many classical algorithms for solving MAX-SAT problems: exact algorithms, stochastic local search algorithms [37,38,39], evolutionary algorithms [40,41], and hybrids of local search and evolutionary algorithms [42,43]. Exact algorithms are often used for small or medium size problems that can be easily verified as satisfied or unsatisfied. The exact algorithms are based on the Davis–Putnam–Logemann–Loveland algorithm (DPLL) [44], an example being the Branch-and-Bound algorithm [45,46] which represents the search space of all possible value assignments to variables as a search tree. Branch-and-Bound explores the branch of the tree and creates new formulas with partial assignments in the internal nodes until the solution is found. The solution is stored in the leaf nodes, which are bound to prevent unnecessary branches. Large size problems use stochastic local search algorithms and evolutionary algorithms which can potentially provide a high-quality solution [42,47].

### 2.3. Quantum Algorithms for Maximum Satisfiability Problem

MAX-SAT is an NP-hard problem and is one of the most widely studied optimization problems in classical algorithms. These NP-hard problems can be potentially solved by quantum algorithms which would offer significant improvements over the classical algorithms, assuming the existence of quantum computers with sufficiently many qubits.

There is some active research to solve the SAT and MAX-SAT problems using the currently available quantum computers, especially the D-wave quantum annealer (QA) systems [48]. The SAT and MAX-SAT are encoded into Quadratic Unconstrained Binary Optimization (QUBO) compatible with the quantum annealer architecture. QUBO is a mathematical class of problems expressed in binary variables as linear or pairwise quadratic terms, which may include constraints.

Practical MAX-SAT problems contain hundreds of variables and terms/clauses which cannot be handled by the currently available quantum computers. Thus, due to the limited number of qubits available, some algorithms suggested reducing the number of qubits. For instance, the quantum cooperative search algorithm for 3-SAT [49] proposed Grover’s search algorithm combined with a classical algorithm that decreases the total number of variables by replacing some qubits with classical bits. However, still, the number of needed ancilla qubits is equal to the number of terms when applied to POS 3-SAT problems.

We propose a new quantum circuit using Grover’s search algorithm, which can be applied to both SAT and MAX-SAT problems with a reduced quantum cost. The main idea is to avoid large Toffoli gates that have high quantum costs and lead to decoherence. Our novel quantum oracle circuit design requires fewer logical qubits to implement the maximum satisfiability problem. This is based on replacing large AND gate collecting results from clauses by a quantum counter that counts the number of satisfied clauses inside the SAT oracle upgraded MAX-SAT oracle. Because modern quantum computers and simulators have a limited total number of qubits, our quantum algorithm allows us to solve larger MAX-SAT problems. However, because of a limited number of qubits, it is not competing with modern software MAX-SAT solvers.

## 3. Definitions and Preliminaries

**Figure 1:**
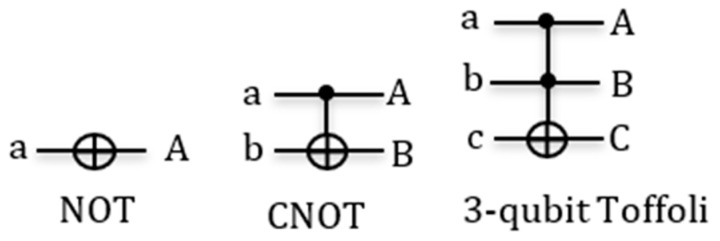
Gate symbol: NOT, CNOT, 3-qubit Toffoli gates.

In this section, we will define some basic concepts related to quantum gates and quantum cost. A few useful gates are shown in Figure 1.

**Definition 1:**  *Reversible gate is* n * n *quantum gate that has* n *input variables and* n *output variables. A quantum gate is reversible if it maps an* n *-input binary vector into a unique* n *-output binary vector. In addition, it is a one-to-one mapping or a permutation of vectors. For example, the* NOT *gate is reversible because if the output is 0, then you know the input must be 1, and vice versa.*

**Definition 2:**  *Controlled-*NOT*(*CNOT*) is a 2-qubit gate, where the first qubit is called control and the second qubit is called target. *CNOT*applies the *NOT*gate on the target qubit when the control qubit is one. The value of the control qubit is not affected. Thus *A=a, B=a⊕b. *The *CNOT*gate is also called the Feynman gate. Using Definition 1, the reader can check that this function is reversible.*

**Definition 3:**  n-*control Toffoli gate consists of*n*-control qubits and one target qubit. The target qubit is inverted if all control qubits are 1. Otherwise, the target qubit is unchanged: *C=ab⊕c. *The values of all control qubits are not changed, thus *A=a, B=b*, etc. This is the universal reversible gate; it realizes AND with c = 0 and NAND with c = 1.*

**Definition 4:**  *Ancilla qubits are extra qubits to allow extra working space during the computation. They are necessary to convert arbitrary Boolean functions to reversible Boolean functions.*

For instance, the Boolean function X = a·b  is not reversible, but function X = a·b⊕c is a reversible gate with c =0.

Although the iterative quantum counter can be built from NOT, CNOT, and multi-qubit Toffoli gates, our design uses Peres gates because the design with Peres gates leads in many cases to substantial circuit cost reduction. Peres gates are built from truly quantum gates CV and CV+ and other Controlled-Nth Root of NOT gates, which requires explaining these gates first.

### 3.1. Nth Root of Not Gate

Mathematically, a quantum gate with n qubit input can be represented as a $2^{n}$ × $\;2^{n}$ unitary matrix. N-th root of NOT gate can be constructed from matrix representation as follows:

$$\sqrt[{n}]{NOT}=\frac{1}{2}\left|\begin{array}{cc} 1+e^{\frac{i\pi }{n}} & 1-e^{\frac{i\pi }{n}}\\ 1-e^{\frac{i\pi }{n}} & 1+e^{\frac{i\pi }{n}} \end{array}\right|.$$

Below given are notations and properties that will be used in the paper to design larger Peres gates: 

V gate $=\sqrt{NOT}$ gate 

$V^{†}$ gate is inverse of V gate. Where $V^{†}$ is called V dagger or conjugate of V.

$W=\sqrt{V}=\sqrt[{4}]{NOT}$

$G=\sqrt{W}=\sqrt[{8}]{NOT}$

VV=NOT

$VV^{†}=I$

WW=V

GG=W

### 3.2. Controlled-Nth Root of NOT Gate

The controlled-Nth root of NOT gate is a 2-qubit gate, where the first qubit is the control, and the second qubit is the target. When the control is one (|1>) then the target qubit calculates the N-th root of NOT gate applied to its input value. Otherwise, with control |0> the target qubit is not changed. The matrix representation of controlled-Nth root of NOT gate is:

$$\;\mathrm{Controlled}-\;\sqrt[{n}]{NOT}=\left|\begin{array}{cccc} 1 & 0 & 0 & 0\\ 0 & 1 & 0 & 0\\ 0 & 0 & \frac{1+e^{\frac{i\pi }{n}}}{2} & \frac{1-e^{\frac{i\pi }{n}}}{2}\\ 0 & 0 & \frac{1-e^{\frac{i\pi }{n}}}{2} & \frac{1+e^{\frac{i\pi }{n}}}{2} \end{array}\right|$$

The inverse of N-th root of NOT gate and controlled-Nth root of NOT gate are constructed from a matrix where the plus and minus signs are reversed.

**Figure 2:**
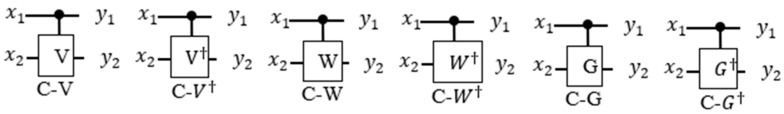
Some symbols for quantum gates of Controlled-nth root of NOT gate and their inverse (†) dagger or conjugate.

Figure 2 shows examples of various controlled-Nth root of NOT gates that we will use in our design of large Peres gates used in counters.

### 3.3. Quantum Cost

Quantum cost of a quantum circuit is the number of elementary quantum gates used to build the circuit. The elementary quantum gates are primitive gates which are 1 × 1 and 2 × 2 reversible gates. The cost of the primitive gates is equal to 1; therefore, the quantum cost is just the number of primitive gates. For illustration, these are three elementary quantum gates that are used to calculate the quantum cost: NOT, controlled-nth root of NOT, and CNOT gates where cost of each gate is equal to 1. (There are some more accurate characterizations of costs of primitive quantum gates [50] but for this paper we use the approximate costs defined as above.) 

**Figure 3:**
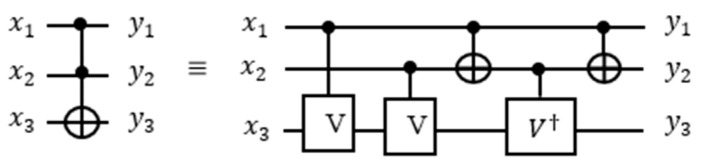
3-bit Toffoli gate represented as controlled-*V*/$\;V^{†}$ and CNOT gates.

Toffoli gate could be built using controlled-nth root of NOT gate [51]. A 3-bit Toffoli gate from Figure 3 has two control qubits and one target qubit and is built from controlled *V*$/V^{†}$ gates and CNOT gates. The quantum cost of the 3-bit Toffoli gate is 5. The generalized formula for quantum cost of m-control Toffoli gate [52] is equal to $2^{m+1}-3$.

### 3.4. Peres Gate

**Figure 4:**
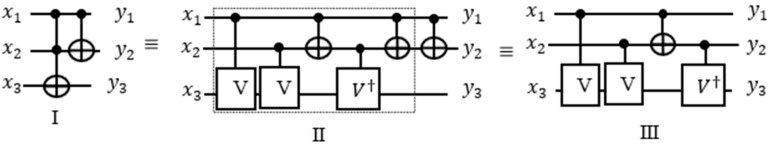
(**I**) 3-bit Peres gate (**II**) decomposed Toffoli gate with CNOT. (**III**) 3-bit Peres gate and its representation using controlled-$V/\;V^{†}$ and CNOT gates.

The Peres gate [53] can be characterized as a sequence of n-Toffoli followed by Feynman (CNOT) gates. For instance, a 3-bit Peres gate consists of a 3-bit Toffoli and a CNOT gates (Figure 4I). When the 3-bit Toffoli and CNOT gates are implemented separately, the cost would be six (Figure 4II). However, the 3-bit Peres gate costs four because the adjacent CNOT gates cancel each other. Thus, the Peres gates are used for quantum cost reduction of quantum circuits and for blocks of the iterative counter in this paper specifically.

**Figure 5:**
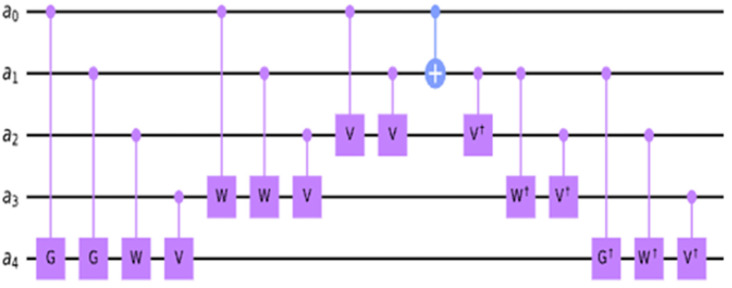
A Peres gate realized on five qubits.

The Figure 4III:

● If $x_{1}$ is 1 and $x_{2}$ is equal to 0 or vice versa, then the transformation applied to $x_{3}$ and one of the V-gate will become active and the other one will be inactive which behaves as the identity. Also, CNOT will become active which produces 1 that will activate $V^{†}$-gate, thus $VV^{†}=I$.
● If both $x_{1}$ and $x_{2}$ are equal to 1, then the transformation applied to $x_{3}$ and two of the V-gate will become active. Also, CNOT will become inactive which produces 0 that will inactivate $V^{†}$-gate, thus VV=NOT.
● If both $x_{1}$ and $x_{2}$ are equal to 0, then no transformation is applied on the gates.
● In general, n-controlled Peres gate consists of n − 1 Toffoli and one CNOT gate. Each n-qubit Peres gate can be built recursively using the n − 1 Peres gate block and a few additional controlled gates. The reader can appreciate this recursive way of building counter blocks of any size by analyzing Figure 5 in which a 4-controlled gate at the right uses the 3-controlled Peres gate in four upper qubits.

**Figure 6:**
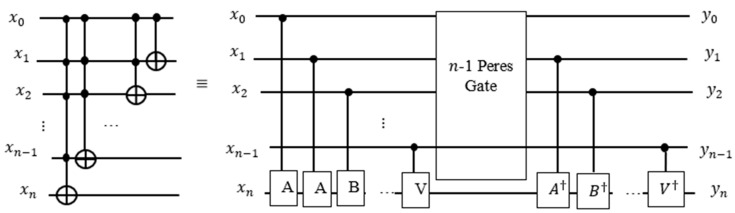
Generalized Peres gate realized on n qubits.

As shown in Figure 5, the 5-qubit Peres gate uses the 4-qubit Peres gate as its sub-circuit. Figure 4 and Figure 5 illustrate that the general formula for the quantum cost of m-controlled Peres gate [54] is equal to $m^{2}$. For a larger design, the Peres gate can be designed as recursive blocks as shown in Figure 6.

### 3.5. Quantum Oracle

An oracle is a black box operation that takes an input and gives an output that is a yes/no decision. A quantum oracle is a reversible circuit that is used in quantum algorithms for the estimation of the value of the Boolean function realized in it. Quantum oracle also has to replicate all input variables on the respective output qubits. If the oracle uses ancilla qubits initialized to |0>, it has to return also a |0> for every ancilla qubit. The classical oracle function is defined as a Boolean function f(x) that takes a proposed solution x of the search problem. If x is the solution, then f(x)=1; If x is not a solution, then f(x)=0. The quantum oracle is a unitary operator O such that:

$$|x\rangle |q\rangle \overset{O}{\to }|x\rangle |q⊕f\left(x\right)\rangle$$

where x is the value in search space, q is a single qubit, the oracle qubit, and ⊕ is the XOR operator (also called the addition modulo 2). A simplified formula of the quantum oracle can be written as:

$$|x\rangle \overset{O}{\to }{\left(-1\right)}^{f\left(x\right)}|x\rangle$$

## 4. Proposed Quantum Algorithm for Maximum Satisfiability

In traditional Grover’s algorithm, oracles are composed of Toffoli and NOT gates; one needs to keep the results of all OR terms for the final AND gate being the decision output of the oracle. The answer to each OR term is stored in a separate ancilla qubit; thus, we need the number of ancilla qubits equal to the number of terms in the function. In Boolean functions involving thousands of terms, this would mean Grover’s oracle needs thousands of ancilla qubits. If there are T terms in a function, we would need T ancilla qubits. For large T, the number of required ancilla qubits becomes unrealistically large, even for future large quantum computers with thousands of logical qubits. Therefore, we present here a novel quantum oracle circuit design that requires $\lceil \log_{2}T\rceil +1$ ancilla qubits when T is not a power of 2 or $\lceil \log_{2}T\rceil +2$ ancilla qubits when T is a power of 2 in order to keep the circuit from growing too large. Our design also improves the overall runtime. For example, in traditional oracles if there are 1,000,000 terms, then we need the same number as 1,000,000 ancilla qubits, but for our design, we need only 21 ancilla qubits. To eliminate the need for ancilla qubits, we make use of the concept of an iterative quantum counter built from blocks, with each block built from controlled Peres gates. We connect one block of the iterative quantum counter after each Toffoli gate representing the OR term of the function POS formula. The satisfiability value of this term controls the block of the counter by activating this block or not. It then increments the count by 1 or 0, depending on the truth value of the OR term. Thus, our quantum counter counts the number of satisfied OR terms in the Boolean function implemented as a POS.

We assign a counter block for each OR term, where the result of the term is used as one of the control qubits of the counter. When the term evaluates to 0, nothing is registered in the counter. When it evaluates to 1, the counter outputs the binary number value+1 to the previously accumulated count value. The use of a quantum counter allows us to send the result from the Toffoli gate representing one OR term to the counter circuit, hence eliminating the need for an ancilla qubit. We can set the function qubit back to 1 by mirroring the Toffoli gate used to compute the result and set the input qubits back to the original by applying NOT gates when appropriate. Our design drastically reduces the number of qubits needed for a function at the cost of replicating Toffoli gates in the POS expression and the costs of the iterative counter.

### 4.1. Grover’s Search Algorithm

Grover’s Algorithm [55] searches an unordered array of N elements to find a particular element with a given property. Grover’s algorithm is often used as a subroutine in other quantum algorithms [56,57,58]. In classical computations, in the worst case, this search takes N queries (tests, evaluations of the classical oracle). In the average case, the particular element will be found in N/2 queries. Grover’s algorithm can find the element in $\sqrt{N}$ queries. Thus, Grover’s algorithm can be used to solve the decision maximum satisfiability k-SAT for every value of k. Grover’s algorithm is a quantum search algorithm, which speeds up a classical search algorithm of complexity $O\left(N\right)\;\mathrm{to}\;O\left(\sqrt{N}\right)$ in the space of N objects, hence Grover gives a quadratic speed up. To solve the optimization problem of finding MAX-SAT with maximum value of k Grover’s Algorithm has to be repeated.

The MAX-SAT contains n variables from the given Boolean function which is used to represent the search space of $N=2^{n}$ elements. To apply the MAX-SAT in Grover’s algorithm, these N elements are applied in a superposition state which is the input to the oracle. If the oracle recognizes an element as the solution, then the phase of the desired state is inverted. This is called the Phase inversion of the marked element. The marked element is a true minterm of function f from the oracle. The true minterm is a product of all variables of function f that evaluates to f = 1. Grover’s search algorithm uses another trick called inversion about the mean (average), which is also known as diffusion operation or amplitude amplification. Inversion about the mean amplifies the amplitude of the marked states and shrinks the amplitudes of other items. The amplitude amplification increases the probability of marked states, so that measuring the final states will return the target solution with a high probability near 1.

**Figure 7:**
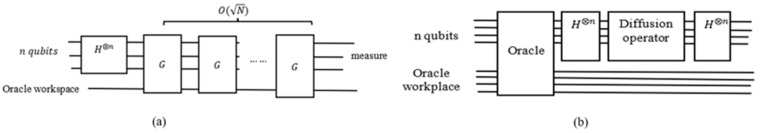
(**a**) Schematic circuit for Grover’s algorithm [55]. (**b**) Grover operator G.

As shown in Figure 7a, the n qubits in the superposition state result from applying a vector of Hadamard gates to initial state $|{0\rangle }^{n}$. Next applied is repeated operator G which is called the Grover Loop. After the iteration of the Grover Loop operator $O\left(\sqrt{N}\right)$ times the output is measured for all input qubits. Oracle can use an arbitrary number of ancilla qubits, but all these qubits must be returned to value |0> inside the oracle. The number of required iterations for Grover operator is: $R\leq \lceil \frac{\pi }{4}\sqrt{\frac{N}{M}}\;\rceil$ where N is number of all search space elements and M is number of solutions. The Grover Loop G is a quantum subroutine which can be broken into four steps as shown in Figure 7b:

1. Phase inversion: apply the oracle. If the oracle recognizes the solution, then the phase of the desired state is inverted
2. Apply the Hadamard transform $H^{⊕n}$ ($H=\frac{1}{\sqrt{2}}\left[\begin{array}{cc} 1 & 1\\ 1 & -1 \end{array}\right]$)
3. Zero state phase shift: Perform the condition phase shift, in which all states receive a phase shift of −1 except for the zero state |0〉.
4. Apply the Hadamard transform $H^{⊕n}$

### 4.2. Quantum Counter

**Figure 8:**
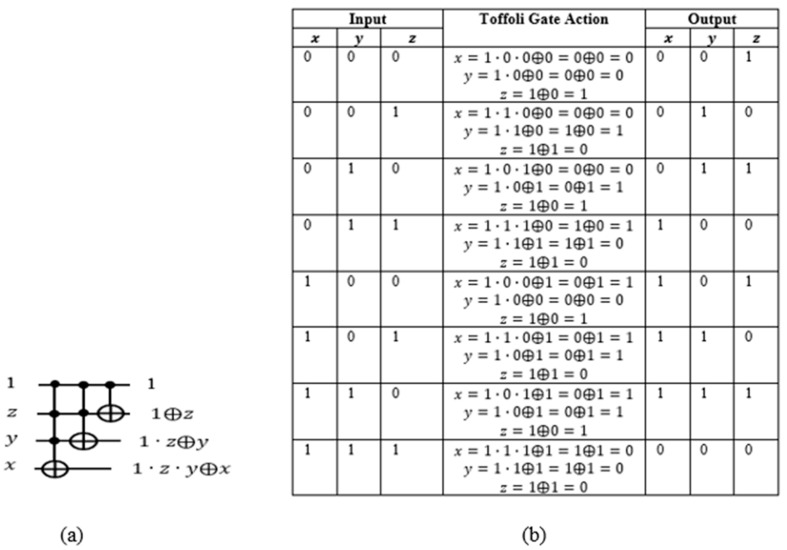
(**a**) Three-qubit quantum counter. (**b**) Analysis of 3-qbit quantum counter block from (**a**).

As described in Section 3.3, the quantum counter block should be constructed from multiple-controlled Peres gates, where the first qubit of the Peres gate is applied a constant 1 with other variables combined, and the Peres gate is then turned into a quantum counter. (This qubit will be next taken from the OR term of the satisfiability formula to activate the counter block realized from Peres gates). For simplicity of explanation, we assume that the counter block is built from Toffoli and CNOT gates, as shown in Figure 8.

Here z is the least significant qubit and x the most significant. The outputs of CNOT and two of the Toffoli gates are 1⊕z, 1⊕z⊕y, and 1·z·y⊕x , respectively. When xyz=000, the first Toffoli gate outputs 1·z·y⊕x=1·0·0⊕0=0⊕0=0 and the second 1·z⊕y=1·0·⊕0=0⊕0=0. The outputs of the qubits y and x are both zeros. The output of the qubit z is 1⊕z=1⊕0=1. Hence the circuit incremented 000 by 1 to 001. Quantum counter circuit indeed outputs the value input+1.

If we connect the first control input of the quantum counter block to a circuit, then the output of the connected circuit (a term of the POS) will either activate or deactivate the counter. When the output of the connected circuit is equal to 1, the output of the counter block is incremented by 1. When the output of the circuit is equal to 0, the output of the counter block is unchanged.

### 4.3. Traditional Oracle for Satisfiability Boolean Function

**Figure 9:**
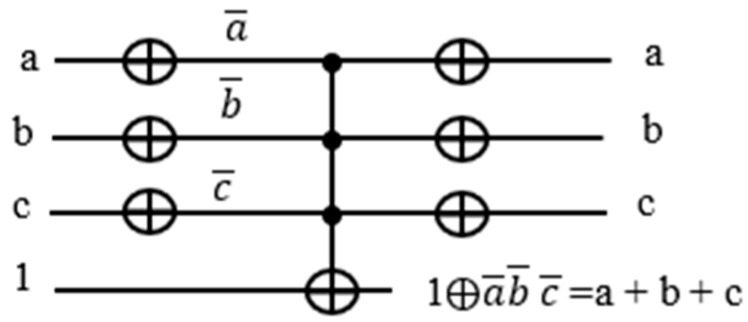
Convert sum term to product term using De Morgan’s law.

To build an OR term using a Toffoli gate, we use De Morgan’s Law to convert the term into a product of the same variables $a+b+c=\bar{\bar{a+b+c}}=\bar{\bar{a}\cdot \bar{b}\cdot \bar{c}}$. With the XOR operation, $1⊕a=\bar{a}$. Hence $a+b+c=\bar{\bar{a}\cdot \bar{b}\cdot \bar{c}}=1⊕\bar{a}\bar{b}\bar{c}.$ The corresponding quantum circuit using a Toffoli gate is shown in Figure 9.

Suppose we have a Boolean function $f\left(a,b,c\right)=\left(a+b+\bar{c}\right)\left(\bar{a}+\bar{b}+c\right)\left(b+c\right)$ from Karnaugh map in Table 1. As one can see in Table 1, there are four which means the solution of the Boolean variables in binaries are (abc = 010, 011, 111, 101), which are satisfied for the Boolean function. Every true minterm in the Karnaugh map from Table 1 is a marked element and potential solution to the Grover Algorithm. However, in one run of Grover’s search algorithm, only one solution is found.

We build a quantum oracle for the Grover’s Loop using Toffoli gates, in which the XOR gate is controlled by the product of variables. We need to first convert the Sum expressions into Products using De Morgan’s Law.

$$a+b+\bar{c}=\bar{\bar{a+b+\bar{c}}}=\bar{\bar{a}\bar{b}\bar{\bar{c}}}=\bar{\bar{a}\bar{b}c}$$

$$\bar{a}+\bar{b}+c=\bar{\bar{\bar{a}+\bar{b}+c}}=\bar{\bar{\bar{\bar{a}}}\bar{\bar{b}}\bar{c}}=\bar{ab\bar{c}}$$

$$b+c=\bar{\bar{b+c}}=\bar{\bar{b}\bar{c}}.$$

**Figure 10:**
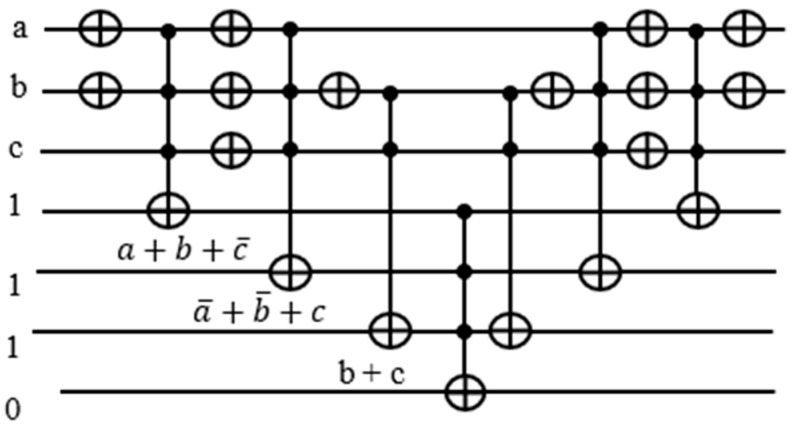
Traditional oracle for Multiple input Toffoli gate used as global AND gate $f=\left(a+b+\bar{c}\right)\left(\bar{a}+\bar{b}+c\right)\left(b+c\right)$.

After building each term with the corresponding product expression, each with an assigned ancilla qubit for the output, we need to put the terms together as the product of the OR terms for the entire function $f\left(a,b,c\right)=\left(a+b+\bar{c}\right)\left(\bar{a}+\bar{b}+c\right)\left(b+c\right).$ Since xyz⊕0=xyz, we use another Toffoli gate controlled by the product of the OR terms XORed with 0. The schematic of the entire circuit for $f\left(a,b,c\right)=\left(a+b+\bar{c}\right)\left(\bar{a}+\bar{b}+c\right)\left(b+c\right)$ is shown in Figure 10:

To set the input qubits and ancilla qubits back to their original states, we mirror all the circuits up to the f(a,b,c) on the right-hand side of the function gate.

Let is define n number for variables and t number for terms then the number of qubits q needed for the oracle is: q=n+t+1.Where 1 is for the OR terms XORed with 0. Notice that we need three ancilla qubits, which is equal to the number of terms. For a function involving thousands of terms, we would need an equal number of ancilla qubits.

### 4.4. Proposed Construction of a Quantum Oracle for MAX-SAT

Our proposed circuit does not require keeping the OR terms for the later calculation of the function. All we need to know is whether each term is satisfied or not, and we pass the result to the counter block assigned to it. Thereafter, we put the ancilla qubit back to the original state 1 by mirroring. Depending on neighboring expressions, there are opportunities to cancel double NOT gates, yet saving the number of gates needed.

**Figure 11:**
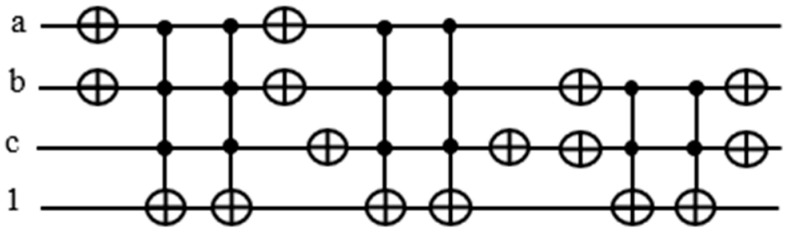
Improved version of the part the oracle $f=\left(a+b+\bar{c}\right)\left(\bar{a}+\bar{b}+c\right)\left(b+c\right)$.

The target output of each Toffoli gate realizing an OR term is used to activate the counter block corresponding to it. In Figure 11, notice that there are two NOT gates adjacent to each other, canceling each other out. Hence, we can remove those gates from our circuit.

**Figure 12:**
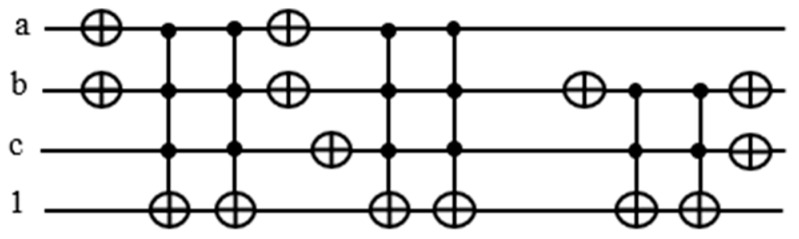
Improved and optimized version of the part the oracle $f=\left(a+b+\bar{c}\right)\left(\bar{a}+\bar{b}+c\right)\left(b+c\right)$.

There are eight NOT and six Toffoli gates in this design in Figure 12 as opposed to 12 NOT and 7 Toffoli gates in the traditional design in Figure 10. The reason we need ancilla qubits in the traditional design is that we need the outputs from the Toffoli gates recorded in the ancilla qubits for counting the number of satisfied terms. By sending the satisfaction result for each term to the quantum counter, we are able to reset the output line back to 1.

**Figure 13:**
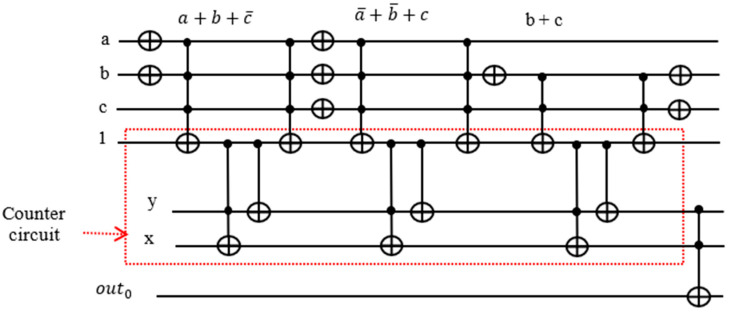
Improved complete oracle using quantum counter.

The count for the number of satisfied terms is output on the xy qubits. In this case, we have three satisfied terms and want to have three as the output expressed as 11 which are expressed as $xy⊕out_{0}$ = xy⊕0 on a Toffoli gate. If the Boolean function f is satisfied, then the outcome $out_{0}$ should be 1. The entire oracle with the function and the iterative counter is shown in Figure 13. We applied this oracle in the Grover search algorithms for R = 2 iterations from this formula: $R\leq \lceil \frac{\pi }{4}\sqrt{\frac{N}{M}}\;\rceil$ where M=4 is the number of solutions in our problem from Table 1, and N = 8 is the number of all search space elements (cells of the Karnaugh map from Table 1). In general, the value of M is calculated using Quantum Counting algorithm [55], but an unsolved problem, the value of M, is taken as 1 to run the Grover iterations R.

**Figure 14:**
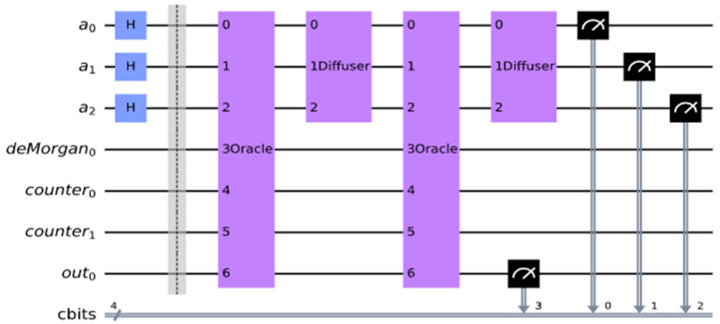
MAX-SAT applied Grover’s search algorithm.$\;f\left(a,b,c\right)=\left(a+b+\bar{c}\right)\left(\bar{a}+\bar{b}+c\right)\left(b+c\right)$.

**Figure 15:**
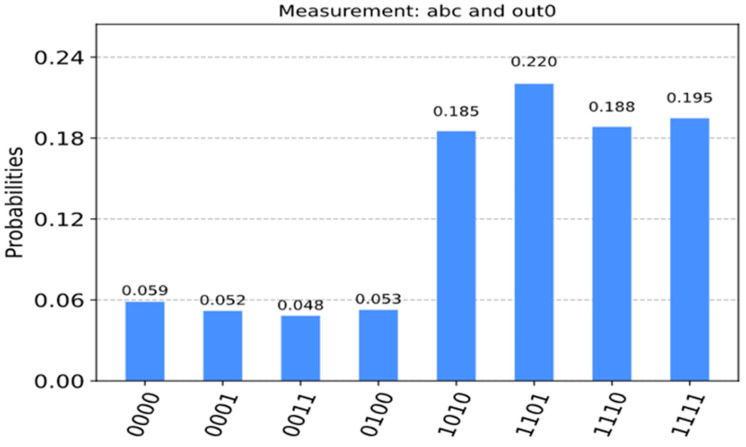
Measurement of the Boolean variables and the outcome of function $f\left(a,b,c\right)=\left(a+b+\bar{c}\right)\left(\bar{a}+\bar{b}+c\right)\left(b+c\right)$.

In Figure 14, we run the circuit on the ‘qasm_simulator’ from QISKIT for 1024 shots (independent runs to obtain high precision probability) for which the circuit produces the correct answers. We measured $a_{0}$, $a_{1}$, $a_{2}$ and $out_{0}$ in Figure 14 where $a_{0}$, $a_{1}$, $a_{2}$ correspond to the Boolean variables, a,b,c, respectively in Figure 13. As can be seen in Figure 15, it illustrates the QISKIT [59] output graphics for the simulated circuit. The measured values with high probability are 1010, 1101, 1110, and 1111, where the most significant qubit is $out_{0}$ which is 1, and the least three significant qubits 010, 101, 110, 111 are all satisfied values for the Boolean function. These solutions correspond to the true minterms from Table 1. For the unsatisfied, the measured values with low probability are 0000, 0001, 0011, and 0100, where the most significant qubit is $out_{0}$ which is 0, and the least three significant qubits 000, 001, 011, 100 are all unsatisfied values for the Boolean function.

As can be seen in Figure 15, the four values 000, 001, 011, and 100 have some value with less probability because of noise created by the simulator. However, we verified the solutions by applying the number of iterations R, and the output from the simulation with high probability 010, 101, 110, and 111 matches the theoretical values, which can be verified manually. We also applied different shots to test, and the results were closely similar, with a high probability for all satisfying values.

### 4.5. Verifying an Unsatisfiable Function

Suppose a function with four OR terms $f\left(a,\;b\right)=\left(a+b\right)\left(\bar{a}+b\right)\left(a+\bar{b}\right)\left(\bar{a}+\bar{b}\right)$ which no assignment of values a and b evaluates the function to 1. We need to first convert the OR terms into Products using De Morgan’s Law and then build the oracle for the given Boolean function.

$$a+b=\bar{\bar{a+b}}=\bar{\bar{a}.\bar{b}}$$

$$\bar{a}+b=\bar{\bar{\bar{a}+b}}=\bar{\bar{\bar{a}}.\bar{b}}=\bar{a.\bar{b}}$$

$$a+\bar{b}=\bar{\bar{a+\bar{b}}}=\bar{\bar{a}.\bar{\bar{b}}}=\bar{\bar{a}.b}$$

$$\bar{a}+\bar{b}=\bar{\bar{\bar{a}+\bar{b}}}=\bar{\bar{\bar{a}}.\bar{\bar{b}}}=\bar{a.b}$$

**Figure 16:**
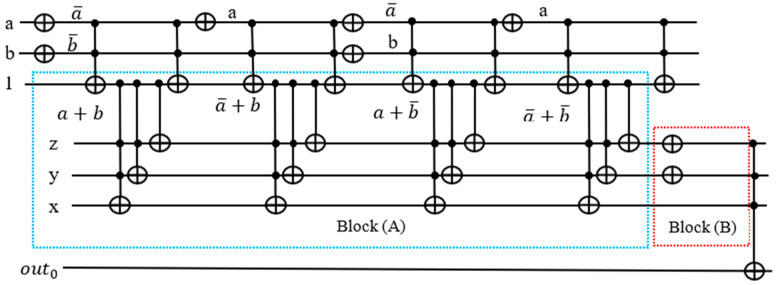
Oracle with counter $f\left(a,\;b\right)=\left(a+b\right)\left(\bar{a}+b\right)\left(a+\bar{b}\right)\left(\bar{a}+\bar{b}\right)$.

The four qubits (1,z,y,x) in block (A) realize the counter, which can count from 0 to 7. We need the last qubit with $out_{0}$ ancilla bit to produce 1 when all terms are satisfied for Grover’s algorithm. Since this function has four terms, to check satisfiability which is the last qubit should be 1, we need to add two NOT gates in the block (B) which makes the last qubit to produce 1 if the Boolean function is satisfied. The function f(a,b) from Figure 16 is not satisfiable, so comparing to a value of 4 in the last gate would not generate any correct solution. Grover’s algorithm will give a few random values that can be verified on the satisfiability formula outside Grover’s Algorithm using function f(a,b). Therefore, we remove the two NOT gates in block (B) to get the maximum satisfied terms of the function.

**Figure 17:**
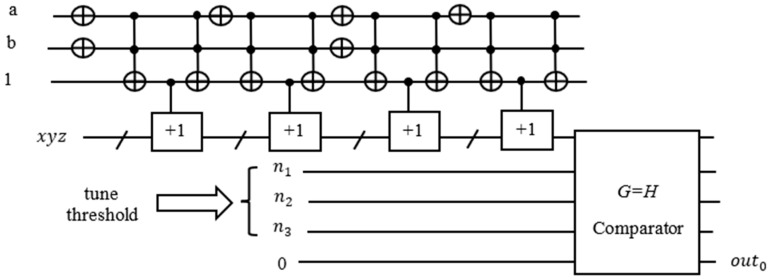
Oracle with counter circuit and threshold with comparator.

In a more general case in Figure 17, we repeat the Grover Algorithm with tuning values of thresholds until equal to counter value xyz. The comparator G=H compares the output from the counter with the threshold value given as constant values $n_{1},n_{1},$ and $n_{3}$. For instance, $f\left(a,\;b\right)=\left(a+b\right)\left(\bar{a}+b\right)\left(a+\bar{b}\right)\left(\bar{a}+\bar{b}\right)$ has 4 terms, we tune the threshold value from 4, 3, 2, and 1 until the condition is met. The value of the counter where the condition is met is the MAX-SAT value. If the condition is met, the ancilla qubit $out_{0}$ will be flipped. It changes the quantum phase of the solution so that the elements that satisfy all constraints are marked. This method of the threshold with comparator is useful to check when the exact number of terms (constraints) are known, which can be checked whether the threshold is equal to the counter value. For instance, if there are 10 constraints in a given function, but it should satisfy a minimum seven constraints, then set the threshold to seven and check if the counter equals to seven. There are applications based on the method of the threshold with a comparator, such as finding the minimum set of support [60].

**Figure 18:**
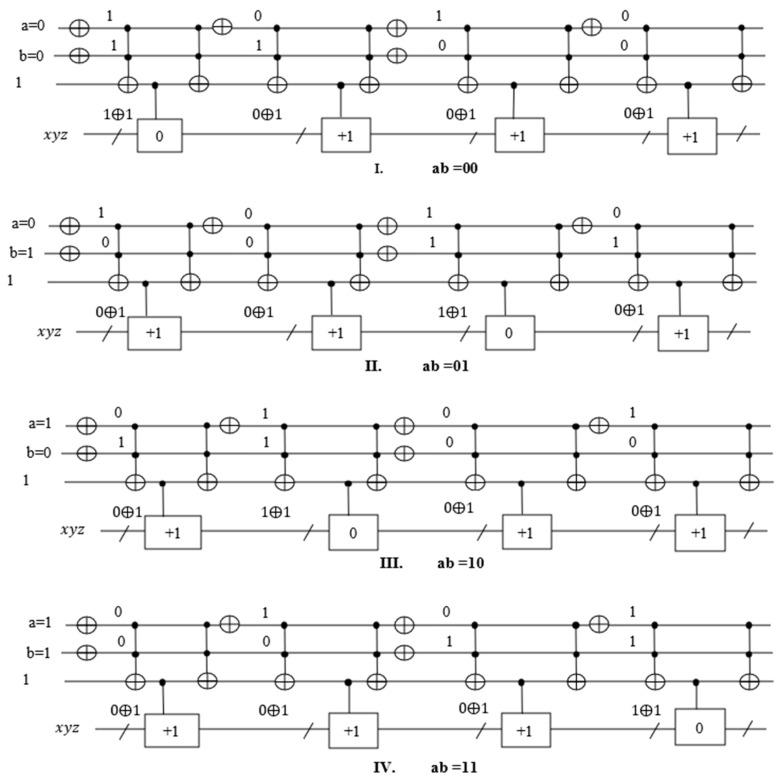
MAX-SAT verification.

**Figure 19:**
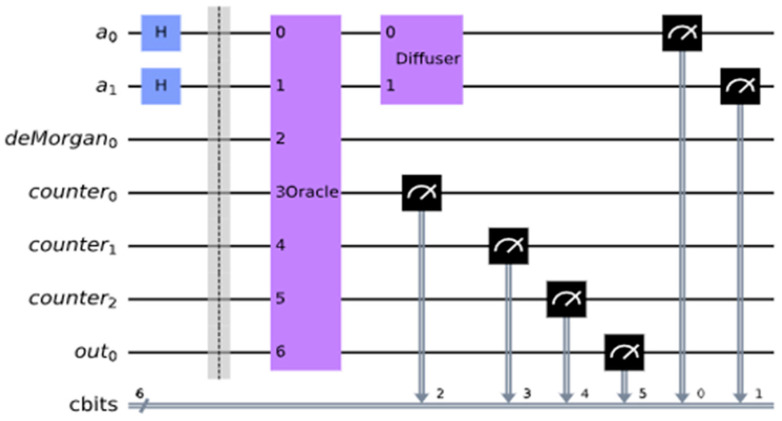
$f\left(a,\;b\right)=\left(a+b\right)\left(\bar{a}+b\right)\left(a+\bar{b}\right)\left(\bar{a}+\bar{b}\right)$ applied Grover’s algorithm.

Every binary vector |a, b〉 of a solution can be verified by running outside of the Grover Algorithm, as can be seen in Figure 18 in which the maximum number of satisfied terms is 3 out of 4. We applied one Grover’s Loop iteration for this oracle to get the MAX-SAT. In Figure 19, we run the circuit on the ‘qasm_simulator’ from QISKIT for 1024 shots.

**Figure 20:**
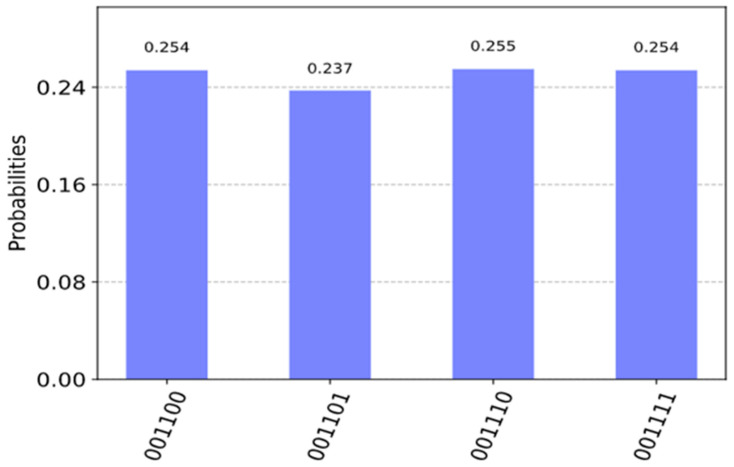
Measurement of $f=\left((a,b,c\right)a+b)\left(\bar{a}+b\right)\left(a+\bar{b}\right)\left(\bar{a}+\bar{b}\right)$.

In Figure 19, we measured the Boolean variables, counter, and output. In Figure 20, the most significant qubit $out_{0}$ always is 0, which means the Boolean function is not satisfied because there are no such binary values for the least two significant qubits 00, 01, 10, and 11, which would satisfy the Boolean function. However, the novelty of our design is that the counter qubits give the maximum numbers of satisfied terms in the Boolean function. The counter qubits are the second, third, and fourth qubits from the most significant qubit, which in this case is 011.

## 5. Calculation of Quantum Cost

### 5.1. Calculation of Quantum Counter Size

In the Table 2 shows the required number of qubits for the quantum counter which each term is not required for one ancilla qubit, but many terms require a few ancilla qubits.

In general, if there are T terms in given Boolean function then the total number of qubits that need for quantum counter is:

● $\lceil \log_{2}T\rceil +1$ ancilla qubits when T is not a power of 2
● $\log_{2}T+2$ ancilla qubits when T is power of 2

**Figure 21:**
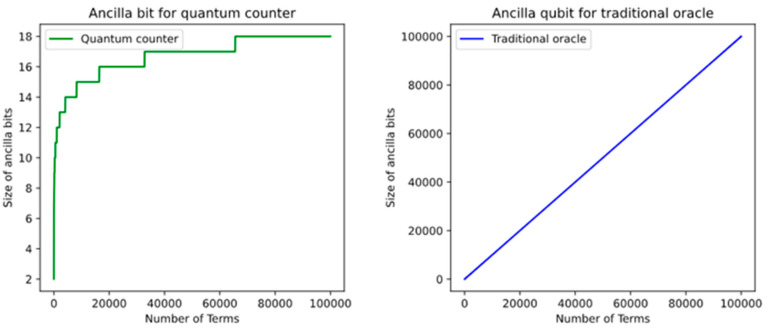
Comparison of required numbers of ancilla qubits for our oracle and the traditional oracle.

As shown in Figure 21, for instance, if there are 100,000 terms, then the number of required ancilla qubits in traditional oracle is 100,000, but in our design, the quantum counter requires only $\lceil \log_{2}T\rceil +1=18$ ancilla qubits. Using the quantum counter, each term is not required for one ancilla qubit, but many terms are required for a few ancilla qubits.

### 5.2. Quantum Cost Calculation for Quantum Counter

Each term in the Boolean function is represented as n-bit Toffoli gate, and the satisfiability result is passed down to the counter. We need as many counter blocks as there are terms in the given POS Boolean function. The counter can be built from Toffoli gates or Peres gates. It is important to have low cost quantum circuits for this high demand for n-bit Toffoli gate. Since the Peres gate is a low-cost quantum circuit, we replaced the Toffoli gates with Peres gates for cost reduction [52]. The formula of quantum cost for m-controlled bits of Peres gate is $m^{2}$ and for Toffoli gate is $2^{m+1}-3$.

**Figure 22:**
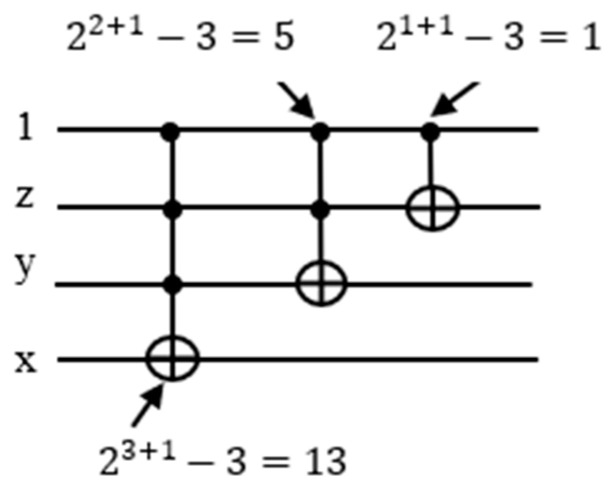
Quantum cost for 3-bit counter.

In Figure 22 a three-qubit counter (3-control qubits) consists of three Toffoli gates which are 3-control, 2-control, and 1-control (CNOT) gates. for each of these Toffoli gates, the quantum cost is calculated separately: $(2^{3+1}-3)+\left(2^{2+1}-3\right)+\left(2^{1+1}-3\right)=28-9=19$. Four-qubit counter consists of four Toffoli gates, and the quantum cost is also calculated separately: $(2^{4+1}-3)+(2^{3+1}-3)+\left(2^{2+1}-3\right)+\left(2^{1+1}-3\right)=60-12=48$.

Thus, we can drive a general formula for the quantum cost of m-bit quantum counter using the Toffoli gate:

$$2^{m+2}-4-3m.$$

The total quantum cost of the quantum counter for each term T is:

(1) $$\mathrm{Peres}\;\cos t=T*m^{2}.$$

(2) $$\mathrm{Toffoli}\;\mathrm{cost}=T*\left(2^{m+2}-4-3m\right).$$

Based on these two Formulas (1) and (2), the Toffoli gate has a higher quantum cost than Peres gate. Thus, we used in our design the Peres gates. As we mentioned before, our final counter uses Peres gates, so we built our oracle using the Peres gate, and it is mapping to the nth root of NOT gates which leads to low quantum cost. The recursive design method from Peres gate was used.

## 6. Variants of SAT Oracles Using Quantum Counter

Following our preliminary work [61], in this section, we discuss some other applications of the quantum counter in variants of satisfiability, such as the product of SOPs SAT.

### 6.1. Oracle for SOPs

**Figure 23:**
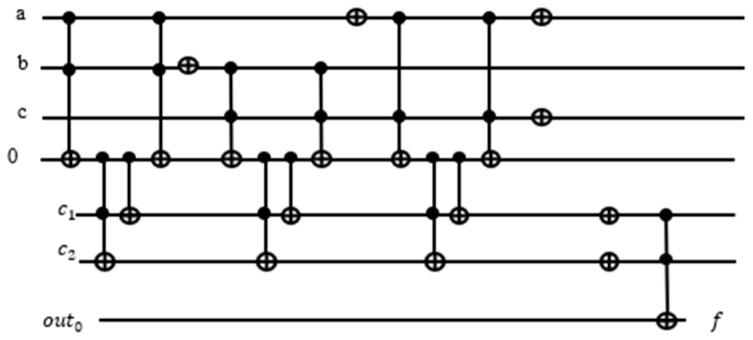
Part of the product of SOP oracle that realizes SOP function $f=ab+b\bar{c}+\bar{a}\bar{c}$.

MAX-SAT can be solved for a Product of any function. In particular, this can be a Product of SOPs. The SOP functions can be implemented with a counter by summing the digits of the counter at the end, using De Morgan’s rule. Each product term is simply a Toffoli gate, and the counter can be checked in a similar way to a regular sum term. Figure 23 presents an example circuit for the function $ab+b\bar{c}+\bar{a}\bar{c}$.

### 6.2. Oracle for Product of SOPs (POSOP SAT)

**Figure 24:**
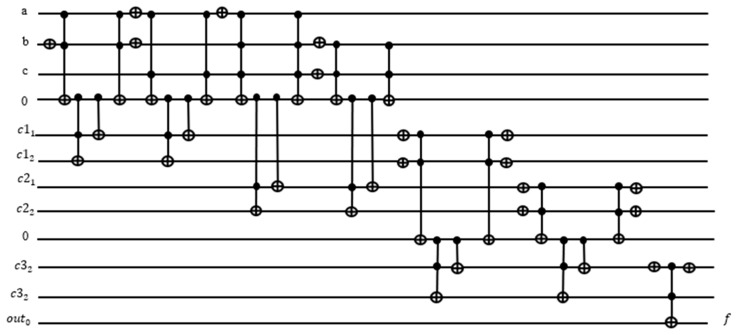
Realization of the Oracle for $f=\left(a\bar{b}+\bar{a}c\right)\left(abc+\bar{b}\bar{c}\right)$, with POSOP SAT.

POSOP functions consist of products of SOP functions. We were not able to find any references to this form of SAT. However, we can take advantage of the fact that every term must be true in a product for the product to be true, and thus we can check against a counter value of the number of terms in order to construct the oracle for POSOP. For example, Figure 24 presents the circuit for function $\left(a\bar{b}+\bar{a}c\right)\left(abc+\bar{b}\bar{c}\right)$.

POSOP circuits are much larger than traditional SOP circuits since an additional counter is required for each SOP term. As such, it may be more advantageous to convert POSOP to a more standard form, such as SOP or POS to be implemented in reversible logic. This depends on a particular problem instance.

### 6.3. Oracle for Exclusive-or-Sum-of-Products (ESOP)

**Figure 25:**
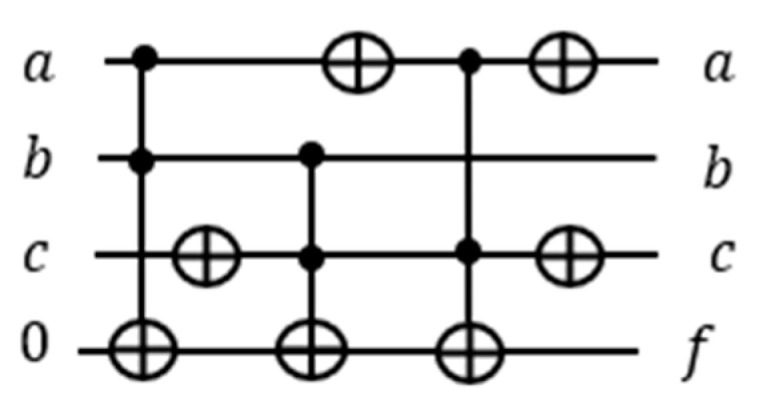
Realization of Oracle $f=ab⊕b\bar{c}⊕\bar{a}\bar{c}$ for ESOP SAT realized in Grover’s Algorithm.

An Exclusive-or-Sum-of-Products (ESOP) form is an exclusive sum (using the ‘⊕’) operator of product terms. There is not much published on ESOP SAT except for [62], although this is an interesting subject. Grover’s Oracle can be trivially applied to ESOP SAT, a problem that has also not been discussed yet. The advantage of ESOP SAT over OR SAT presented in the previous section is that ESOP SAT can be realized without the need for a large AND gate or a counter. Since every product in the EXOR sum can be implemented as a Toffoli gate, SAT with ESOP can be formulated with just the input qubits and one output qubit. For example, given a function such as $ab⊕b\bar{c}⊕\bar{a}\bar{c}$, we can implement Grover’s Oracle, as shown in Figure 25.

## 7. OR Satisfiability Problems for Electronic Design Automation

In this section, we will show that many EDA (Electronic Design Automation) problems can be reduced to SAT and MAX-SAT. In the most general case, the Satisfiability Decision Function problem is formulated as an arbitrary binary-valued-input, binary-output, and single-output function. For instance, a product of sums of literals, (the literals are variables negated or not), EXOR of products of literals, and product of sums of products of literals. These functions are created by transforming some natural language or mathematical decision problems, such as, for instance, cryptographic puzzles. The question is to find out for which values of variables the formula for SAT or MAX-SAT is satisfied. In some problems, one has to find all solutions; in some other problems we look for just one solution or only some solutions. For all these variants, we have some freedom to modify Grover’s Algorithm, and/or call it several times with modified oracles [60].

Below we will systematically formulate several satisfiability types of problems, starting from the simplest ones. We concentrate on problems that have applications in EDA. Each of these basic problems below can have in addition several variants related to specific applications. Given is a product of terms, each term being a Boolean sum of literals, and each literal being a Boolean variable or its negation. We are interested in the following problems.

**Problem 1**  (**Satisfiability**)**:** *Answer Yes if there exists a product of literals that satisfies all terms or No if such a product does not exist. Give the solution as a set of literals.*

**Problem 2**  (**Optimization of the Generalized Petrick function**)**:** *Find a product with the minimum number of literals that satisfies all terms or prove that such a product does not exist*.

**Problem 3**  (**Optimization of the Generalized Petrick function-nonnegated literal variant**)**:** *Find such a product of literals that satisfies all terms and in which a minimum number of literals is not negated or prove that no such product exists. (The not negated literals will also be called positive literals). In particular, the Petrick Function is positive unate, which it means has only positive literals*.

**Problem 4**  (**MAX-SAT**)**:** *Find such set of literals that satisfies the maximum number of terms*.

**Problem 5**  (**Tautology Checking**): *Verify whether a function is a Sum of Product Form is a Boolean tautology. Function* F *is a tautology (all input combinations are 1) when its negation* $\bar{F}$ *is not satisfiable (all combinations are 0).*

In some variants of these problems, depending on a particular application, we can look for all solutions, all optimal solutions, some optimal solutions, or for a single optimal solution. The central role of the Problem 1 is well-established in computer science. All NP-complete combinational decision problems are equivalent to the Satisfiability Problem [63]. Many reductions of practically important problems to other above problems were shown, including problems from VLSI Design Automation, especially in logic design and state machine design. SAT and MAX-SAT also have many applications in logistics, scheduling, AI, and robotics. Ashenhurt/Curtis Decomposition of Boolean functions can be done in an algorithm that repeatedly applies Satisfiability [64]. Generalized Ashenhurst/Curtis Decomposition was also realized by building a complex oracle for Grover’s Algorithm based on the mathematics of Partition Calculus [65]. These SAT-like problem formulations are also of fundamental importance in many algorithms for Boolean minimization, factorization, and multi-level design. The set covering problem is reduced to the minimization of Petrick Function. The reductions of many practically important NP-hard combinatorial optimization problems can also be found in the literature. For instance, the minimization of the Sum of Products Boolean functions can be reduced to the Covering Problem [66] and Covering Problem can be further reduced to the Petrick Function Optimization Problem (PFOP) [67]. Many other problems, like test minimization, can also be reduced to the Covering Problem [66,68]. The problems of Partial Satisfiability and its applications are discussed by K. Lieberherr [69]. Many other reductions to the formulated above problems are discussed in [63,70]. Paper [71] discusses the reduction of three-level NAND circuits, TANT, to the covering-closure problem solved similarly to SAT. A similar problem of the synthesis of networks from negative gates uses the same reduction [72]. A design automation system [73] was created, in which many problems were first reduced to the few selected “generic” combinatorial optimization problems. These problems include some of the problems listed above.

The problem of minimization of Finite State Machines includes: (1) the Maximum Clique Problem and (2) the problem of finding the minimum closed and a complete subgraph of a graph (Closure/Covering Problem) [71]. The first of these problems, (1), can be reduced to the Petrick Function Optimization Problem (PFOP). The problem of optimum output phase optimization of PLA [74] can be reduced to PFOP. The second problem, (2), can be reduced to the Generalized Petrick Function Optimization Problem (GPFOP), introduced above and illustrated below. Many other problems, like AND/OR graph searching [75], were reduced to the Closure/Covering Problem.

A number of problems (including Boolean minimization [76], layout compaction, and minimization of the number of registers in hardware compilation can be reduced to the Minimal Graph Coloring Problem. Regular layout problems can be reduced to SAT [77]. The Minimal Graph Coloring can be reduced to the Problem of Finding the Maximum Independent Sets, and next the Covering Problem (Maghout algorithm). The Problem of Finding the Maximum Independent Sets can be reduced to PFOP. The PFOP is a particular case of the GPFOP. The role and importance of Tautology and conversion methods from SOP to POS and vice versa in logic design are well known. These problems can also be solved using SAT.

Concluding on OR SAT. In theory, every NP problem can be polynomially reduced to SAT and also to OR 3-SAT. But this is not practical. Many problems can be reduced to graph coloring or maximum clique problems that can be in turn reduced to satisfiability problems.

As we see now, many problems can be solved with quadratic speedup using future quantum computers. A hybrid classical/quantum computer based on Grover tuned to solve variants of SAT problems of various types would be a tremendous asset to all these problems [60].

## 8. Conclusions

We have designed a novel quantum oracle circuit that requires a logarithmically reduced number of qubits for solving SAT and MAX-SAT problems. The oracle circuit uses the iterative quantum counter circuit, which replaces the ancilla qubits of a global large AND gate for traditional oracle design. Our design showed a significant reduction overall in the number of qubits in Grover’s search algorithm for MAX-SAT. Also, our design calculates the quantum measurable number of the maximum satisfiable OR terms for unsatisfiable Boolean functions. We also compared using Peres and Toffoli gates in terms of quantum cost, where the Peres gates built from truly quantum primitives provide lower quantum costs. Finally, we tested and showed two examples on the IBM QISKIT simulator [23] that provided the expected results. We presented other Variants of SAT oracles that can be designed for the oracle circuit using the quantum counter. Also, we discussed many other potential problems in the area of EDA that can be reduced to SAT and MAX-SAT such that the oracle can be constructed the quantum counter idea.

Suppose one wants to calculate the number of satisfied true minterms for a SAT or MAX-SAT problems. This corresponds to the number of ones in certain Boolean functions. This type of problem is solved using the Quantum Counting Algorithm [14], which in turn is based on Quantum Phase Estimation. Also, many other quantum algorithms use oracles with large AND gate at the output. We plan to work on finding solutions to these problems. The obvious improvement and generalization will be that the yes/no solutions will be extended to solutions for non-solvable problems where the answer will be given to tell how far we are from the solution by creating the “MAX versions” of the problems instead of the current “YES/NO” versions.

## Figures and Tables

**Table 1:** Karnaugh map of POS for the Boolean function $f\left(a,b,c\right)=\left(a+b+\bar{c}\right)\left(\bar{a}+\bar{b}+c\right)\left(b+c\right)$.

ab\c	0	1
**00**	0	0
**01**	1	1
**11**	0	1
**10**	0	1

**Table 2:** Quantum counter size; total qubits for counter.

Number of Terms (Clauses)	Total Qubits for Quantum Counter
2	$\lceil \log_{2}T\rceil +2$ = 3
3	$\lceil \log_{2}T\rceil +1$ = 3
4	$\lceil \log_{2}T\rceil +2$ = 4
5…7	$\lceil \log_{2}T\rceil +1$ = 4
8	$\lceil \log_{2}T\rceil +2$ = 5
9…15	$\lceil \log_{2}T\rceil +1$ = 5
16	$\lceil \log_{2}T\rceil +2$ = 6
17…31	$\lceil \log_{2}T\rceil +1$ = 6
32	$\lceil \log_{2}T\rceil +1$ = 7
33… 63	$\lceil \log_{2}T\rceil +1$ = 7
64	$\lceil \log_{2}T\rceil +2$ = 8
65…127	$\lceil \log_{2}T\rceil +1$ = 8
128	$\lceil \log_{2}T\rceil +2$ = 9
129…255	$\lceil \log_{2}T\rceil +1$ = 9
256	$\lceil \log_{2}T\rceil +2$ = 10
257…511	$\lceil \log_{2}T\rceil +1$ = 10
…	…
…	…
*T*	$\{\begin{array}{ll} \lceil \log_{2}T\rceil +1, & if\;\;T\;\mathrm{is}\;\mathrm{not}\;\mathrm{power}\;\mathrm{of}\;2\\ \lceil \log_{2}T\rceil +2, & if\;\;T\;\mathrm{is}\;\mathrm{power}\;\mathrm{of}\;2 \end{array}$

## Data Availability

Not applicable.
